# Portable, Rapid, and Cost-Effective Smartphone-Based Colorimetric Quantification of Total Lactones in *Andrographis paniculata*

**DOI:** 10.3390/ph19071110

**Published:** 2026-07-18

**Authors:** Sutasinee Apichai, Suphakorn Katib, Teerapat Ouirungroj, Thanawat Pattananandecha, Kanokwan Kiwfo, Fumihiko Ogata, Naohito Kawasaki, Kate Grudpan, Chalermpong Saenjum

**Affiliations:** 1 Office of Research Administration, Chiang Mai University, Chiang Mai 50200, Thailand; thanawat.patt@cmu.ac.th (T.P.); k.kanokwan11@gmail.com (K.K.); 2 Research Center for Innovation in Analytical Science and Technology for Biodiversity-Based Economic and Society (I-ANALY-S-T_B.BES-CMU), Multidisciplinary Research Institute (MDRI), Chiang Mai University, Chiang Mai 50200, Thailand; suphakorn2801@gmail.com (S.K.); phc28pipe@gmail.com (T.O.); kate.g@cmu.ac.th (K.G.); 3 Department of Pharmaceutical Sciences, Faculty of Pharmacy, Chiang Mai University, Chiang Mai 50200, Thailand; 4 Faculty of Pharmacy, Kindai University, 3-4-1 Kowakae, Higashi-Osaka, Osaka 577-8502, Japan; ogata@phar.kindai.ac.jp (F.O.); kawasaki@phar.kindai.ac.jp (N.K.); 5 Antiaging Center, Kindai University, 3-4-1 Kowakae, Higashi-Osaka, Osaka 577-8502, Japan; 6 Department of Chemistry, Faculty of Science, Chiang Mai University, Chiang Mai 50200, Thailand

**Keywords:** total lactone, andrographolide, smartphone, colorimetry, image processing, in-field/on-site analysis

## Abstract

**Background/Objectives**: *Andrographis paniculata* is listed in the Thai National List of Essential Medicines. The Thai Herbal Pharmacopoeia specifies the required contents of total lactones and andrographolide for the quality control of the aerial parts of *A. paniculata*. Effective pre-harvest quality control throughout the cultivation period is essential to ensure compliance with these quality standards. In this study, we aimed to develop a portable smartphone-based colorimetric method for the determination of total lactone content, thereby facilitating in-field quality control of *A. paniculata* raw materials. **Methods**: Methanol was used as the extraction solvent, and the analytes were extracted using a simple procedure derived from the United States Pharmacopeia concept. The colorimetric reaction was based on a charge-transfer reaction between the α,β-unsaturated γ-lactone moiety and 3,5-dinitrobenzoic acid, producing a red-purple product in a microwell plate. The delta-green intensity of the reaction product was captured as an andrographolide equivalent using a smartphone camera and quantified through digital image processing with a custom-developed mobile application. **Results**: A linear working range of 15–100 µg/mL was exhibited with limits of detection and quantification of 4.9 and 15.0 µg/mL, respectively. The practical applicability of the developed method was evaluated using *A. paniculata* samples and compared with the conventional spectrophotometric method. The results showed excellent agreement between the two methods, with a correlation coefficient of 0.9986. **Conclusions**: The results of this study suggest that this portable and rapid method is feasible for in-field/on-site analysis to facilitate pre-harvest quality control throughout the cultivation period, ensuring that harvested *A. paniculata* materials comply with established quality standards for plant-derived natural active pharmaceutical ingredients (NAPIs). The proposed method has the potential to promote sustainable production and responsible resource utilization by improving the quality of herbal raw materials intended for the manufacture of herbal medicines and dietary supplements, in line with UN-SDG #12 and #3.

## 1. Introduction

Currently, the demand for medicines, including pharmaceuticals, herbal medicines, herbal products, and dietary supplements, is steadily increasing, whereas natural resources are becoming increasingly limited. In addition, growing reports of adverse effects associated with herbal medicines and concerns that overharvesting may lead to the extinction of endangered species have increased awareness of resource management, particularly for medicinal plants, to ensure their safe, sustainable, and optimal use [1]. The *Guidelines on Good Agricultural and Collection Practices* [2] *for Medicinal Plants* established by the World Health Organization cover cultivation, harvesting, post-harvest handling, and legal standards, including site selection, plant identification, quality control, patents, and benefit sharing. These involve one of the strategies based on in-field analysis of medicinal plants to enhance the availability of efficacious plant-based natural pharmaceutical active ingredients (NAPIs) [3].

In-field/on-site crop analysis is a key component of precision and sustainable agriculture, which aims to optimize production by supporting sustainable crop management [4]. Techniques such as spectral sensors, hyperspectral and multispectral imaging, thermal imaging, electrochemical sensors, and bio/chemical sensors are employed to enable rapid assessment [5]. Simultaneously, in-field/on-site crop analysis techniques continue to advance, overcoming challenges related to analytical accuracy, field equipment availability (e.g., cost and ease of use), and data management. Smartphone-based portable devices for in-field/on-site crop analysis have attracted considerable attention as modern technologies supporting smart farming approaches [6,7]. Most of these devices have been developed by integrating optical sensors, particularly fluorometric and colorimetric reactions, for rapid and cost-effective detection [8,9,10,11].

*Andrographis paniculata* (Burm. f.) Nees, commonly known as “King of Bitters,” has been widely used in traditional medicine throughout Asia. The plant is distributed in India, Taiwan, China, and many regions of Southeast Asia and tropical/subtropical Asia, including Vietnam, Sri Lanka, Thailand, Indonesia, Laos, Malaysia, Cambodia, and the Caribbean Islands. It has also been reported to spread across various edaphic zones in China, America, the West Indies, and Christmas Island [12]. It has long been used as an antiviral, antimalarial, antipyretic, analgesic, antivenom, hepatoprotective, antioxidant, antidiabetic [13], immunostimulatory, antimicrobial [14], and anti-inflammatory agent [15]. Additionally, it has exhibited cardiovascular [16], neuroprotective, and anticancer activities [17]. These properties are associated with NAPIs, particularly diterpene lactones present in both free and glycosidic forms, including andrographolide (AG), 14-deoxyandrographolide, 14-deoxy-11,12-didehydroandrographolide, and neoandrographolide as major constituents [18]. From past to present and continuing into future trends, extensive scientific research has been conducted on their molecular and biochemical aspects to improve their applications, as well as to investigate the biosynthesis of their active constituents [19,20,21].

In practical applications, guidelines and standards have been established to control the quality of *A. paniculata*, particularly its lactone content, from raw materials to finished products. Pharmaceutical companies use United States Pharmacopeia (USP) standards to evaluate *A. paniculata* products and ensure that they meet the required specifications for consumers [22,23,24]. For raw materials, the acceptance criterion is that the total content of the diterpene lactones AG, neoandrographolide, 14-deoxy-11,12-didehydroandrographolide, and andrograpanin must be no less than 1.0% on a dried basis. According to the monograph established by the World Health Organization (WHO) [25], the material should contain no less than 6% total diterpene lactones, calculated as AG. According to the Thai Herbal Pharmacopoeia (THP), *A. paniculata* (commonly known as “Fa Thalai” or “Fa Thalai Chon”) is required to contain no less than 6.0% (*w*/*w*) total lactones, calculated as AG [26]. Several analytical methods have been used for the quality control of lactone content [27,28,29]. Selecting an appropriate method is crucial and mainly depends on the established analytical objectives. In general, High-Performance Liquid Chromatography (HPLC) [30,31,32,33] and spectrophotometric methods [34,35,36,37,38,39,40] are commonly employed. In addition, Fourier transform infrared (FTIR) spectroscopy has also been reported [41]. However, these methods are relatively complicated for routine quality assessment throughout the entire process, from cultivation and harvesting to *A. paniculata* production, due to limitations related to instrument availability, operational costs, and analyst expertise. Table S1 summarizes some methods and their key features. To meet the requirements of on-site analysis in real-world applications, considerable efforts have been made to develop simple analytical techniques. For example, a predictive model for estimating the lactone content in *A. paniculata* has been reported [42]. Nevertheless, there remains considerable scope for investigating and developing alternative simple analytical approaches.

This work aimed to develop a smartphone-based portable, rapid, and cost-effective approach for NAPI content quantification, with a particular focus on total lactones in *A. paniculata*, an economically important medicinal plant widely used in pharmaceuticals, herbal medicines, and dietary supplements. To support in-field analysis, a simple sample preparation method based on extraction, a colorimetric analytical procedure using a smartphone as the detector, and automatic evaluation through a custom-built mobile application were developed.

## 2. Results and Discussion

### 2.1. Visible Spectra of Total Lactone Expressed as AG with 3,5-Dinitrobenzoic Acid

**Figure 1:**
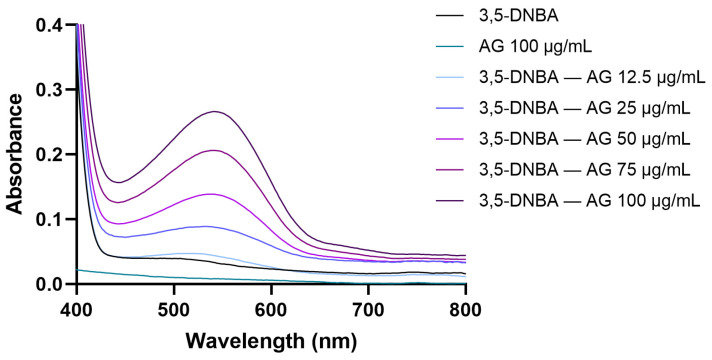
Visible absorption spectra of the red-purple product between 3,5-DNBA and different concentrations of AG.

*A. paniculata* contains diterpenoid lactones, with AG and 14-deoxy-11,12-didehydroandrographolide as the major constituents. These compounds possess an α,β-unsaturated γ-lactone moiety [18,31], which is responsible for the Kedde reaction [43]. Specifically, the α,β-unsaturated γ-lactone moiety reacts with 3,5-dinitrobenzoic acid (3,5-DNBA) under alkaline conditions to form a red-purple product (the proposed structure and reaction mechanism have been reported in [43] to exhibit a maximum absorbance at 541 nm. Under basic conditions, the lactone is activated, enabling interaction with the electron-deficient dinitrobenzoate reagent, which is commonly described as a charge-transfer interaction. AG was used as a representative lactone standard. These lactones react with 3,5-DNBA to produce a red-purple product. The spectrum showed maximum absorption at 536 nm, as shown in Figure 1. Absorbance increased proportionally with increasing AG concentration.

### 2.2. Optimization of Smartphone-Based Colorimetric Analytical Conditions

The relationship between the color intensity of the red-purple product and the concentration of AG was investigated using an image-processing technique. The Kedde reaction was performed, and the resulting images were acquired using a controlled illumination box to ensure consistent lighting conditions throughout the analysis. Although there is no evidence that the Kedde reaction is inherently photosensitive or requires protection from light, the use of the illumination box minimizes the influence of ambient lighting, thereby improving the reliability and reproducibility of smartphone-based colorimetric measurements. Green color is complementary to the red-purple in the color space. The G-value would exhibit the highest intensity within the RGB color mode. The experimental results supported this through the linear calibrations obtained by plotting the change in color intensity relative to the blank (Δ intensity) vs. concentration of AG (for 0–100 µg/mL AG: ΔG = 0.828[AG] + 0.649, R^2^ = 0.997; for green: ΔR = 0.337[AG] + 2.272, R^2^ = 0.981, ΔB = 0.267[AG] + 1.881, R^2^ = 0.978). The green color exhibited the highest sensitivity, as indicated by the fact that it has the steepest slope and excellent linearity, demonstrating that it is the most suitable channel for the quantitative determination of AG.

#### 2.2.1. Concentration of KOH

**Figure 2:**
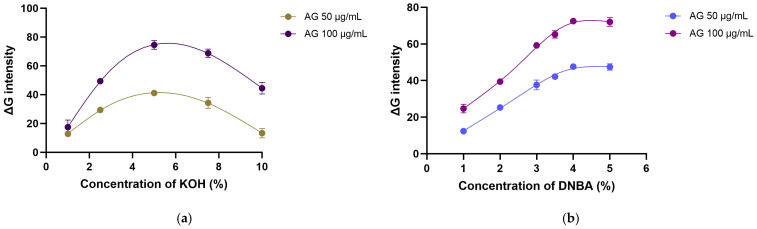
Effect of (**a**) KOH concentration and (**b**) 3,5-dinitrobenzoic acid (3,5-DNBA) concentration on the relative green intensity of the red-purple product formed by the Kedde reaction with andrographolide (AG; 50 and 100 µg/mL). KOH concentrations (1, 2.5, 5, 7.5, and 10%) were evaluated at a fixed 3,5-DNBA concentration of 4%, whereas 3,5-DNBA concentrations (1, 2, 3, 3.5, 4, and 5%) were evaluated at a fixed KOH concentration of 5%.

Alkaline conditions were critical because they enhanced the electrophilicity of the lactone and the nucleophilicity of 3,5-DNBA. The KOH concentration was varied at 1, 2.5, 5, 7.5, and 10%, while the concentration of 3,5-DNBA was fixed at 4%. The color product increased by increasing the KOH concentration up to 5%. Above 5% KOH, a decrease in color product formation was observed from Δ green intensity, as shown in Figure 2a. This reduction was possibly due to the effect of excess alkalinity on the degradation of the lactone ring of AG [44]. A KOH concentration of 5% was selected as the optimum alkaline condition.

#### 2.2.2. Concentration of DNBA

The optimum concentration of 3,5-DNBA for color formation was investigated. 3,5-DNBA concentrations of 1, 2, 3, 3.5, 4, and 5% were varied in an alkaline solution containing a fixed concentration of 5% KOH. The production of the color product resulting from the reaction of 50 and 100 µg/mL AG with the mixed reagents was measured. The color product increased with increasing 3,5-DNBA concentration until it reached a stable level at 4% 3,5-DNBA, indicating a suitable concentration (see Figure 2b).

#### 2.2.3. Temperature

The effect of temperature on color product formation was also investigated. The incubation temperature varied from 25 to 60 °C. Figure S1 demonstrates that incubation temperatures ranging from 20 to 40 °C produced comparable color changes in the reaction product. Therefore, sample analysis can be performed reliably within this temperature range. For on-site applications, the ambient temperature in plantations of *A. paniculata* in most tropical regions typically falls within this range, indicating that the proposed method is suitable for field-based analysis without requiring strict temperature control.

#### 2.2.4. Incubation Time

Monitoring of the color product was performed to determine the optimum detection time. The results indicated that the color product was stable for 3–6 min, followed by progressive color fading during prolonged incubation. The sensitivity, expressed as the slope of the relationship between the change in G intensity and AG concentration, is shown in Figure S2, the highest sensitivity was observed at the incubation time of 3–6 min.

#### 2.2.5. The Stability of Mixed Reagents

Kedde’s reagent is commonly prepared freshly before analysis because the chromophore formed in alkaline medium exhibits limited stability. For cost-effective and rapid on-site analysis, the stability of the prepared mixed reagents was essential. KOH and 3,5-DNBA were mixed and stored under ambient light at room temperature. At different time intervals, aliquots of the mixture were taken to evaluate reagent stability by comparing the sensitivities (slopes of the calibration graphs). The results indicated that the color production efficiency remained stable, exhibiting consistent sensitivity for at least 2 h after preparation of the mixed reagent, as shown in Figure S3.

### 2.3. Investigation of Analytical Characteristics 

The analytical characteristics of the developed approach, performed according to Section 3.2 and Section 3.3, were investigated. The analytical characteristics exhibited a working range of 15–100 µg/mL, with a calibration equation of ΔG = 0.822[AG] + 1.129 (*R*^2^ = 0.995). The limit of detection (LOD, 3.3σ) and limit of quantification (LOQ, 10σ) values were 4.93 and 15.0 μg/mL, respectively, where σ was calculated as the standard deviation of the intercept divided by the slope of the calibration graph. The key features of the developed method are compared with those of previous studies in Table S1.

Four concentrations of blind samples, prepared from the AG standard, were determined according to Section 3.2 and Section 3.3. The average percentage recoveries (%recovery) ranged from 96 to 102%, indicating acceptable accuracy. Relative standard deviation (%RSD) values within each day ranged from 1 to 6% for repeatability, while the %RSD values for intermediate precision were 2–4%. Table S2 shows the % recoveries and the %RSD values.

The effects of interfering compounds, including alkaloids (quinine and caffeine), flavonoids (rutin and catechin), and phenolic compounds (caffeic acid, gallic acid, ellagic acid, and tannic acid), were investigated. The expected interfering compounds were spiked into AG to obtain final concentrations of 5, 12.5, and 25 µg/mL, while the AG concentration was fixed at 25 µg/mL. The mixtures were determined for AG concentration, and percentage recoveries were evaluated. Table S3 shows the obtained % recoveries of the mixtures. Mixtures containing quinine, caffeine, caffeic acid, ellagic acid, and tannic acid showed no significant interference, with percentage recoveries of 91–98%, 90–100%, and 92–108%, respectively. In contrast, gallic acid and catechin exhibited tolerance limits of 25 and 12.5 µg/mL, respectively, in the presence of 25 µg/mL AG. In practice, the *w*/*w* ratios of gallic acid and catechin to AG are well below the acceptable tolerance limits [38,45,46,47]. Their presence does not significantly interfere, as indicated by the percentage recoveries obtained.

### 2.4. Performance of Total Lactone Extraction

#### 2.4.1. The Effect of Solvent

Methanol and ethanol are commonly used for the extraction of lactones, including AG, while some studies have also reported the use of acetone, ethyl acetate, and other solvents [48]. The USP recommends methanol as the extraction solvent for *A. paniculata*. In addition, previous studies have demonstrated that methanol provides high extraction efficiency for andrographolide and related diterpene lactones [22,49]. The effect of solvents on colorimetric detection was investigated. AG standard solutions (50 and 100 µg/mL) were prepared in various solvents, including MeOH, EtOH, ACN, acetone, and EtOAc, and then reacted with the reagents according to Section 3.2. Figure S4 shows the Δ green intensity of the color products obtained from AG standards prepared in different solvents. The results showed that AG dissolved in MeOH and EtOH reacted with the reagent to produce a red-purple product in proportion to the concentration. However, the green intensity of the product in MeOH was higher than that in EtOH. In contrast, the color solutions obtained from AG dissolved in ACN, acetone, and EtOAc appeared light violet, deep violet, and gray, respectively, and showed no proportionality to the concentration. MeOH was selected for the extraction of *A. paniculata* samples because it produced a more favorable red-purple color response.

#### 2.4.2. Sample–Solvent Ratio

Several extraction techniques have been reported for lactone extraction from *A. paniculata*, including maceration [34,38], mechanical mixing (shaking or vortexing) [47], sonication [39], reflux or Soxhlet extraction [22], microwave-assisted extraction [50], and supercritical fluid extraction [51]. In this work, the shaking technique was selected because the study aimed to develop a simple and rapid analytical method using minimal chemicals and readily available miniaturized devices to provide convenient access. The solid-to-liquid ratio, one of the factors affecting extraction, was investigated. According to the USP [22], WHO [25], and THP [26] guidelines, the samples consisted of the aerial parts; therefore, both leaves and stems were used. The samples of *A. paniculata* leaves and stems were weighed at 0.5, 1.0, 1.5, 2.0, and 3.0 g. Each sample was extracted in triplicate with 10 mL of MeOH by shaking at 140 rpm for 15 min, followed by filtration through a nylon syringe filter with a pore size of 0.22 μm. The extract solutions were then determined for total lactone content according to Section 3.2. The results showed that a sample-to-MeOH ratio of 1:10 (1 g of *A. paniculata* in 10 mL MeOH) provided the highest yield, as indicated by the total lactone content shown in Figure S5.

#### 2.4.3. Extraction Time

The extraction time was optimized. The samples of *A. paniculata* leaves and stems (1 g each) were extracted with 10 mL MeOH by shaking at 140 rpm for different extraction times (3, 5, 10, 15, and 20 min), followed by filtration through a nylon syringe filter with a 0.22 μm pore size. Each sample was prepared in triplicate. The total lactone content of the extract solutions was then determined according to Section 3.2. Figure S6 shows the total lactone contents obtained at different extraction times. The low concentrations of total lactones present in the stems were extracted in similar amounts over the 5–20 min extraction period. In contrast, the extraction time clearly affected the concentrations of total lactones obtained from the leaves up to 15 min, after which the extracted concentrations began to stabilize. Therefore, an extraction time of 15 min was selected.

#### 2.4.4. Correlation with the United States Pharmacopeia and the National Formulary

The efficiency of the optimized extraction procedure was investigated and compared with the extraction procedure recommended in the USP–NF [22]. Twelve *A. paniculata* samples were extracted using the optimized extraction procedure (as described in Section 3.4). For the USP procedure, the same samples were extracted according to Section 3.5 and subsequently filtered. Each sample was prepared in triplicate. The extract solution was then filtered through a 0.22 μm nylon syringe filter and its total lactone content was determined according to Section 3.2. The yields obtained from the developed procedure agreed with those obtained from the USP procedure. This demonstrated the feasibility of the developed extraction procedure.

### 2.5. Application to Real Samples

**Figure 3:**
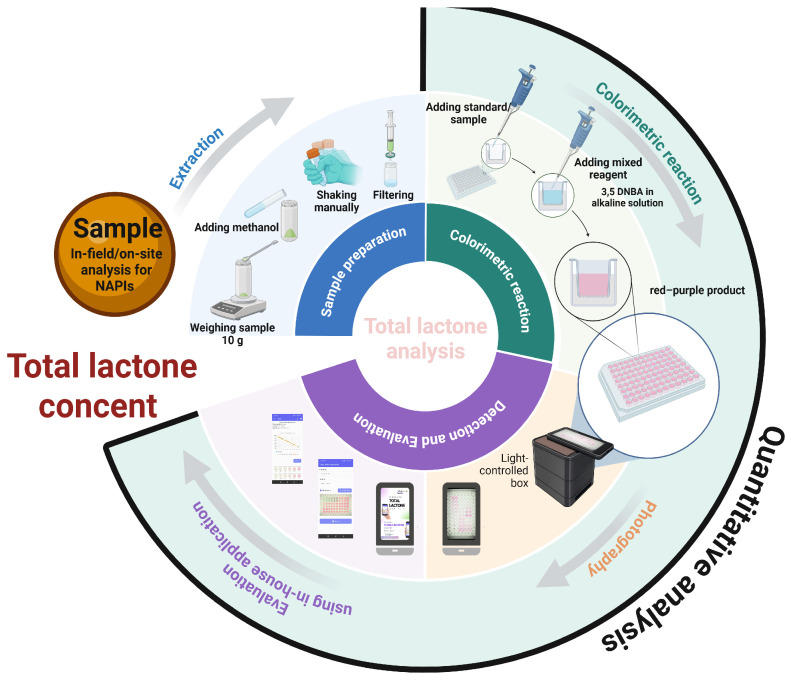
Schematic illustration of the overall workflow for the developed smartphone-based colorimetric assay for total lactone determination in *A. paniculata*. Created in BioRender. Apichai, S. (2026) https://BioRender.com/qlbxn0z. (accessed on 15 June 2026).

**Figure 4:**
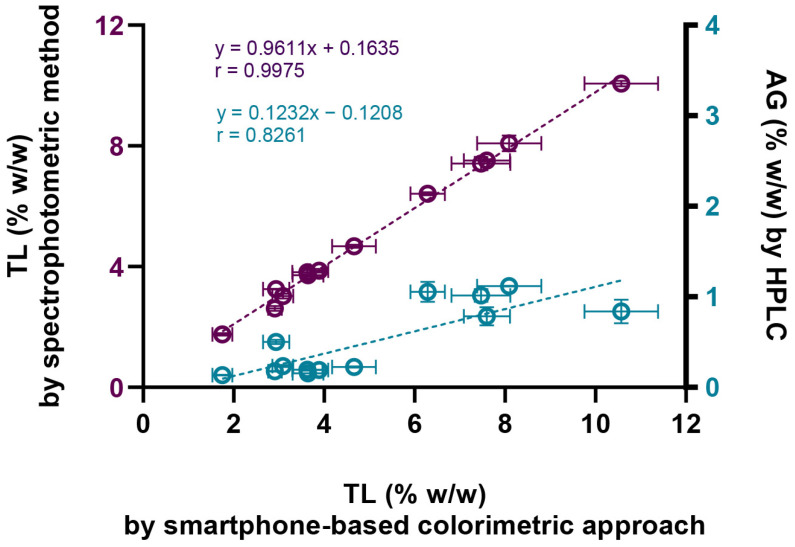
Correlation of TL contents, expressed as AG, in *A. paniculata* samples (determined using the developed approach) with those obtained by the spectrophotometric method (left *y*-axis) and with those determined by HPLC (right *y*-axis).

To demonstrate the real-world application, thirteen *A. paniculata* samples, including leaves and stems analyzed separately, were extracted; then total lactone content was determined using the developed approach (as illustrated by the workflow in Figure 3) and spectrophotometric analysis at 536 nm, while AG content was quantified by HPLC (Section 3.6). Each sample was performed in triplicate. The samples were diluted 100- and 200-fold to fall within the linear working range. The results obtained using the developed approach showed good agreement with those of the spectrophotometric method, with a correlation coefficient of 0.9975, as shown in Figure 4. Linear regression yielded a slope of 0.9611 (95% CI: 0.9162–1.006) and an intercept of 0.1715 (95% CI: −0.08455 to 0.4276), indicating no statistically significant proportional or constant bias between the smartphone-based assay and the reference spectrophotometric method. Bland–Altman analysis was additionally performed to evaluate agreement between the smartphone-based and spectrophotometric methods. The analysis showed a mean bias of 0.028% total lactones with 95% limits of agreement from −0.374% to 0.430%, demonstrating good agreement between the two methods. Using the pharmacopeial acceptance criterion of 6.0% total lactones, the smartphone-based method correctly classified all 13 samples, corresponding to a decision accuracy of 100% (five true positives, eight true negatives, no false positives, and no false negatives). Additionally, a correlation was observed between smartphone-based colorimetric total lactone measurement and AG content determined by High-Performance Liquid Chromatography, indicating a similar trend between the two methods.

To demonstrate the ability to overcome matrix effects, four samples were spiked with a series of andrographolide (AG) standard solutions, and the total lactone contents, expressed as andrographolide equivalents (AGE), were determined in triplicate using the smartphone-based colorimetric method with external calibration and standard addition, compared with the spectrophotometric method. As shown in Table 1, no statistically significant differences were observed among the three methods at the 95% confidence level (*p* > 0.05). This demonstrated that the proposed method will strongly support United Nations Sustainable Development Goal (SDG) 12 (Responsible Consumption and Production) by promoting efficient natural resource management and reducing waste through early herbal quality assessment before harvesting in the future. It minimizes agricultural losses by reducing the disposal of substandard raw materials. It promotes sustainable production through quality control and traceability throughout the medicinal plant supply chain, thereby enhancing transparency across the production process. The approach further promotes the production of high-quality herbal medicines with consistent quality, aligning with SDG 3 (Good Health and Well-Being).

## 3. Materials and Methods

### 3.1. Chemicals

All chemicals were of analytical grade. Andrographolide, caffeine, caffeic acid, catechin, 3,5-DNBA, and rutin were purchased from Tokyo Chemical Industry Co., Ltd. (Tokyo, Japan). Gallic acid and quinine were obtained from Sigma-Aldrich (St. Louis, MO, USA). Acetone, acetonitrile, ethyl acetate, ethanol, methanol, and potassium hydroxide were obtained from RCI Labscan Ltd. (Bangkok, Thailand).

### 3.2. The Developed Analytical Procedure for Total Lactone

A smartphone-based colorimetric method for total lactone analysis was developed using a microscale colorimetric reaction in a 96-well microplate, with the smartphone serving as the detector. Briefly, 150 μL of a set of standards in duplicate and the extract samples were added to each well and reacted with 150 μL of the reagent mixture, consisting of equal volumes of 4% (*w*/*v*) 3,5-DNBA and 5% (*w*/*v*) KOH. The reaction mixture was then incubated for 3 min to produce a colored product. After the completed reaction, the microplate was placed inside a light-controlled box under fixed illumination provided by a Godox LEDM150 LED light source (Godox Photo Equipment Co., Ltd., Shenzhen, China; 1029 lx). The camera-to-plate distance was maintained at 19 cm throughout image acquisition. Images were captured using the default camera application of the Redmi Note 13 5G (Xiaomi Corporation, Beijing, China). Automatic exposure (AE), autofocus (AF), and automatic white balance (AWB) were enabled, whereas high dynamic range (HDR), flash, digital zoom, and image filters were disabled to ensure consistent imaging conditions. During method development, the green-channel intensity in the Red–Green–Blue (RGB) color model was analyzed from digital color photographs using ImageJ software (version 1.54g. A custom-built application developed in our laboratory was used for the analytical characterization of the developed approach and real-sample analysis. A calibration graph for total lactone evaluation was constructed by plotting the concentration of AG against the change in Δ green intensity.

### 3.3. Mobile Application Handling

**Figure 5:**
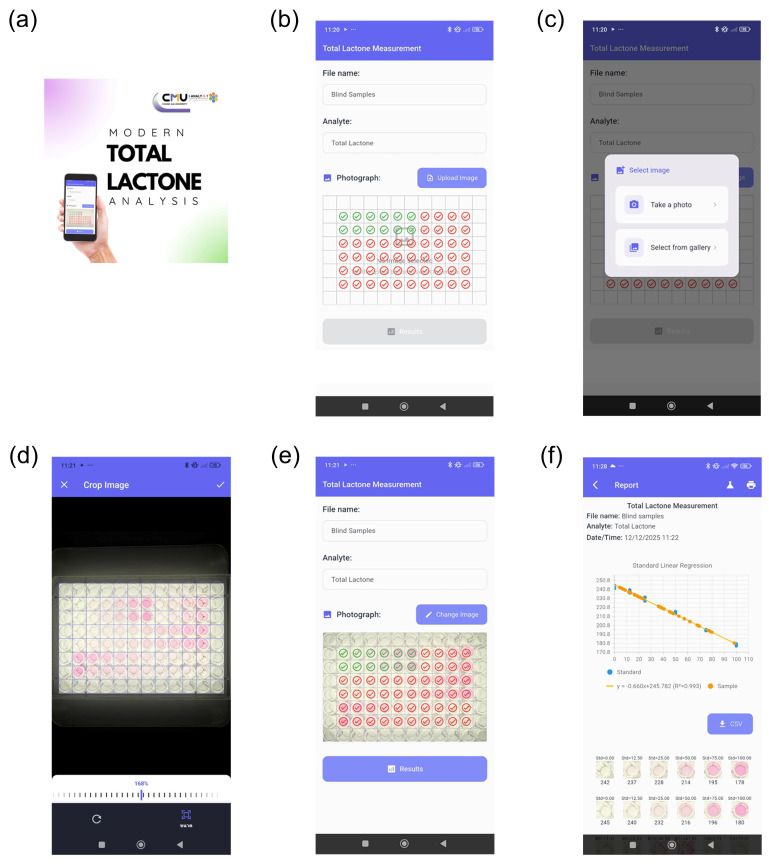
User interface of the MODERN TOTAL LACTONE ANALYSIS application illustrating the analysis workflow: (**a**) application icon and home screen; (**b**) sample information and plate-layout configuration; (**c**) image upload by capturing a photograph or importing one from the gallery; (**d**) layout adjustment; (**e**) completion of the required information and initiation of analysis by pressing the Results button; and (**f**) display of the results.

A mobile app (MODERN TOTAL LACTONE ANALYSIS, Figure 5a) for the Android platform was modified in-house from previous work [52] to evaluate total lactone concentrations. The application imports photographs acquired according to the procedure described in Section 3.2. One photo accommodates a set of standards in duplicate (0, 12.5, 25, 50, 75, and 100 µg/mL) and samples in 48 wells of a 96-well microplate. Positions of the standards were fixed in the 96 wells (see Figure 5b). So were the samples. The app features a dedicated button for importing photographs, allowing users to either capture an image directly using the camera or select one from the device’s gallery (Figure 5c). The photograph can be rotated and zoomed to align with the designated outline, as shown in Figure 5d. The total lactone evaluation is performed by pressing the “Result” button, as shown in Figure 5e. The region of interest (ROI) corresponding to each well was manually selected by tapping the center of the reaction well. The application then extracted the mean green-channel intensity from a predefined circular ROI. For each acquired image, the application automatically generated a calibration curve using the standard solutions included in the same image. Subsequently, the total lactone concentration of each sample was calculated and displayed on the application interface (Figure 5f), with the results available for export in CSV format. To evaluate the repeatability of the image-processing algorithm, the same photograph containing AG standards and sample wells was analyzed repeatedly (n = 10) using the developed application. The calculated total lactone concentrations exhibited excellent repeatability, with a relative standard deviation (RSD) of less than 4%, indicating that the image-processing algorithm provided highly reproducible analytical results.

### 3.4. The Development of On-Site Total Lactone Extraction

*A. paniculata* samples (1 g each) were extracted with 10 mL of MeOH by hand shaking for 15 min and then allowed to stand until the solids settled and the layers separated. The extract solution was filtered through a 0.22 μm nylon syringe filter for analysis.

### 3.5. The Reference Method for Sample Extraction

The reference method for total lactone extraction was performed according to the procedure recommended by the USP–NF [22]. The *A. paniculata* samples (2 g) were extracted with 50 mL of MeOH by refluxing for 15 min. After cooling to room temperature, the supernatant was decanted and retained. The extraction procedure was repeated three additional times, and all supernatants were combined. The combined extract was filtered and concentrated under vacuum, and the final volume was adjusted to 50.0 mL with MeOH.

### 3.6. High-Performance Liquid Chromatography (HPLC)

AG analysis was performed using an Agilent 1200 series HPLC system (Agilent Technologies, Santa Clara, CA, USA). The separation was achieved using a Kinetex^®^ Polar C18 column (150 × 4.6 mm, 2.6 µm, 100 Å; Phenomenex, Torrance, CA, USA) maintained at 30 °C. The mobile phase consisted of 0.1% acetic acid in deionized water and 0.1% acetic acid in acetonitrile at a ratio of 70:30 (*v*/*v*). The flow rate was set at 0.5 mL/min, and the injection volume was 10 µL. Detection was carried out at 230 nm using a UV detector.

## 4. Conclusions

In this work, a smartphone-based portable, rapid, and cost-effective colorimetric quantification method for total lactones in *Andrographis paniculata* was developed. Lactones could be extracted from *A. paniculata* as a model plant (stem and leaves) with methanol, using simple, cost-effective, and easily available apparatus and shaking by hand. Four samples could be handled simultaneously for 15 min. The obtained extract could be quantified for total lactone by Keddy’s reaction using a micro-pipette and a 96-well plate platform. The operation was carried out with microliter scales. A smartphone with the in-house-developed app was used for detection and evaluation. The operation handled 24 samples in duplicate by using a set of six duplicated standards within 10 min. Combining the extraction part with the quantitation steps provides an alternative method for performing assays of total lactones in real samples, as demonstrated by the results being in agreement with those of conventional spectrophotometry. The developed method has various advantageous features (instrument cost, simplicity of procedure, and suitability for on-site use) compared to previous reports (Table S1). This indicates the feasibility of applying the method for on-site screening for primary quality control. Investigations involving samples from different plantation eco-systems, including planting cycles and harvest seasons/conditions, are in progress. Such screening will be very beneficial in places where limited budget is available, especially areas with fewer opportunities. The method can be operated without the need for electrical power at the site and will be significantly useful in some places, such as Thailand, as *A. paniculata* is officially listed as one of the herbal medicines in the “National List of Herbal Medicines”, a part of the “National List of Essential Medicines” (NLEM) of Thailand [53]. The developed method supports the United Nations Sustainable Development Goals, particularly #12 (Responsible Consumption and Production) and #3 (Good Health and Well-Being).

## Figures and Tables

**Table 1:** Comparison of total lactone contents in *A. paniculata* samples determined by external calibration and standard addition using the developed approach and the spectrophotometric method (*n* = 3).

Sample	Concentration of Total Lactone Expressed as AG (%*w*/*w*)		
	The Developed Approach (Smartphone-Based Colorimetric Approach)		The Spectrophotometric Method
	External Calibration	Standard Addition	
1	7.6 ± 0.5	7.5 ± 0.1	7.52 ± 0.05
2	6.3 ± 0.4	6.4 ± 0.2	6.41 ± 0.05
3	3.6 ± 0.3	3.8 ± 0.3	3.71 ± 0.04
4	4.7 ± 0.5	4.7 ± 0.2	4.68± 0.06

## Data Availability

The data presented in this study are available on reasonable request from the corresponding author.

## Supplementary Materials

The following supporting information can be downloaded at: https://www.mdpi.com/article/10.3390/ph19071110/s1, Figure S1: Effect of incubation temperature (25, 33, 40, 50, and 60 °C) on the relative green intensity of the red–purple charge-transfer product formed between andrographolide (AG; 50 and 100 µg/mL) and the optimized reagent concentrations (4% 3,5-DNBA and 5% KOH). Error bars represent the standard deviation (SD) of three independent measurements (*n* = 3).; Figure S2: Effect of incubation time (1–20 min) on the sensitivity, expressed as the slope of the calibration graph obtained for andrographolide (AG; 0–100 µg/mL) using the optimized reagent concentrations of 4% 3,5-DNBA and 5% KOH. Error bars represent the standard deviation (SD) of three independent measurements (*n* = 3).; Figure S3: Stability of the mixed reagents over the mixing time, evaluated in terms of sensitivity, defined as the slope of the calibration graph for andrographolide (AG; 0–100 µg/mL) using the optimized reagent concentrations of 4% 3,5-dinitrobenzoic acid (3,5-DNBA) and 5% KOH. Error bars represent the standard deviation (SD) of three independent measurements (*n* = 3).; Figure S4: Relative green intensities of the red–purple product formed from the reaction between AG (50 and 100 µg/mL) dissolved in different solvents and the optimized reagent concentrations of 4% 3,5-dinitrobenzoic acid (3,5-DNBA) and 5% KOH. Error bars represent the standard deviation (SD) of three independent measurements (*n* = 3).; Figure S5: TL contents, expressed as AG, in leaves and stems of the *A. paniculata* samples extracted using different sample-to-solvent ratios (0.5, 1, 1.5, 2, and 3 g in 10 mL MeOH).; Figure S6: TL contents, expressed as AG, in leaves and stems of the *A. paniculata* samples extracted with different extraction times (3, 5, 10, 15, and 20 min).; Table S1: Methods for quantitation of lactones in *A. paniculata*: comparison of key features.; Table S2: Accuracy and precision (repeatability and intermediate precision) for total lactone.; Table S3: Average percentage recoveries obtained from AG standard solutions (25 µg/mL) spiked with interfering compounds.

## Abbreviations

The following abbreviations are used in this manuscript:

AG	Andrographolide
DBNA	Dinitrobenzoic acid
NAPIs	Natural pharmaceutical active ingredients
TL	Total lactone
