# Supplementary material for: Portable, Rapid, and Cost-Effective Smartphone-Based Colorimetric Quantification of Total Lactones in Andrographis paniculata
Source: Pharmaceuticals (Basel). 2026 Jul 18;19(7):1110. doi: 10.3390/ph19071110 (PMC13415145; doi:10.3390/ph19071110)
Supplement: Supplementary file 1 [file pharmaceuticals-19-01110-s001.zip › pharmaceuticals-4409865-supplementary.pdf]

pharmaceuticals

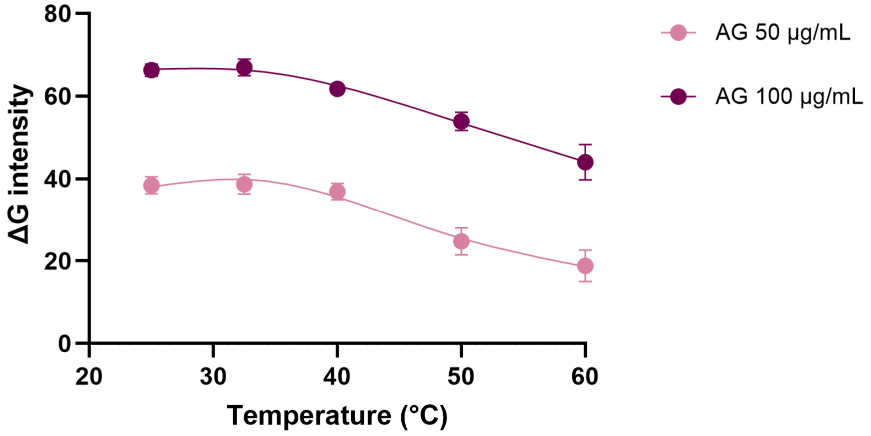

Figure S1. Effect of incubation temperature (25, 33, 40, 50, and 60 °C) on the relative green intensity of the red–purple charge-transfer product formed between andrographolide (AG; 50 and 100 µg/mL) and the optimized reagent concentrations (4% 3,5-DNBA and 5% KOH). Error bars represent the standard deviation (SD) of three independent measurements (n = 3).

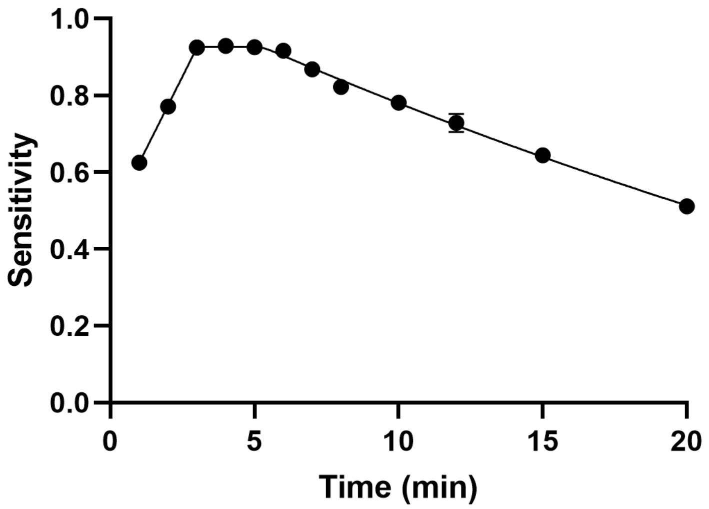

Figure S2. Effect of incubation time (1–20 min) on the sensitivity, expressed as the slope of the calibration graph obtained for andrographolide (AG; 0–100 µg/mL) using the optimized reagent concentrations of 4% 3,5-DNBA and 5% KOH. Error bars represent the standard deviation (SD) of three independent measurements (n = 3).

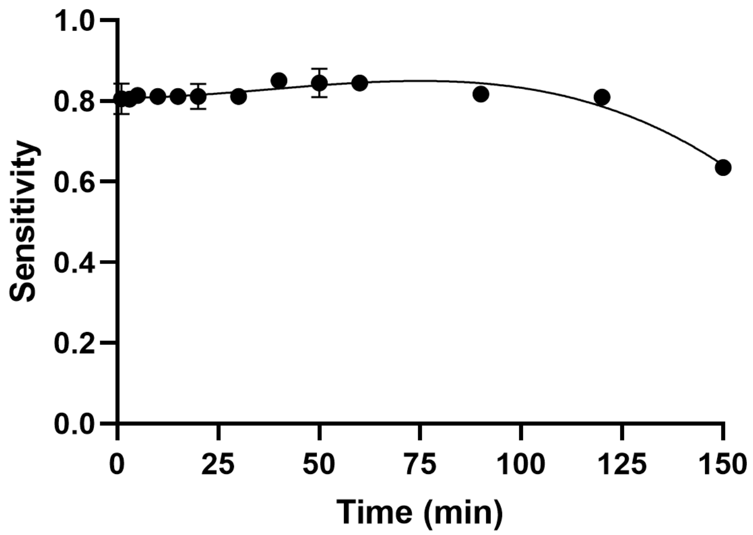

Figure S3. Stability of the mixed reagents over the mixing time, evaluated in terms of sensitivity, defined as the slope of the calibration graph for andrographolide (AG; 0–100 µg/mL) using the optimized reagent concentrations of 4% 3,5-dinitrobenzoic acid (3,5-DNBA) and 5% KOH. Error bars represent the standard deviation (SD) of three independent measurements ($n = 3$).

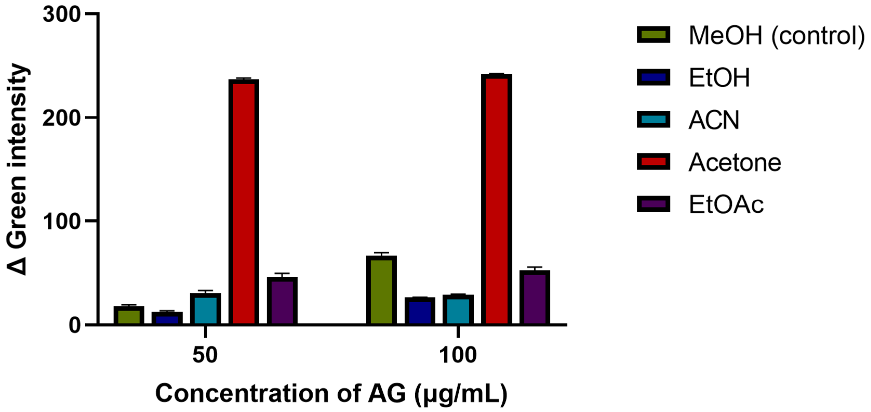

Figure S4. Relative green intensities of the red–purple product formed from the reaction between AG (50 and 100 µg/mL) dissolved in different solvents and the optimized reagent concentrations of 4% 3,5-dinitrobenzoic acid (3,5-DNBA) and 5% KOH. Error bars represent the standard deviation (SD) of three independent measurements ($n = 3$).

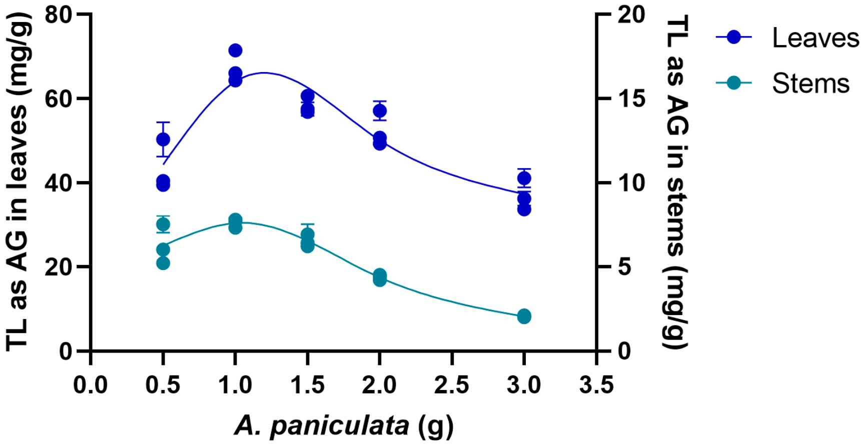

Figure S5. TL contents, expressed as AG, in leaves and stems of the *A. paniculata* samples extracted using different sample-to-solvent ratios (0.5, 1, 1.5, 2, and 3 g in 10 mL MeOH).

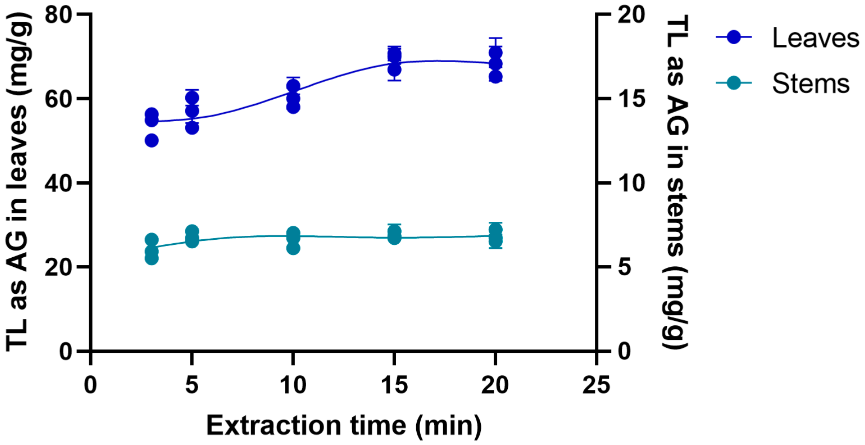

Figure S6. TL contents, expressed as AG, in leaves and stems of the *A. paniculata* samples extracted with different extraction times (3, 5, 10, 15, and 20 min).

1

Table S1 Methods for quantitation of lactones in *A. paniculata*: comparison of key features.

Method	Condition to extract the analyte from the sample					Technique for quantitation										Overall			Reference
	Procedure steps	Amount of raw material	Solvent	Temperature	Time	Technique	Reagent/ chemicals	Detector	Linear range	LOD	LOQ	Analysis time	sample throughput/h	Amount of sample	Consumption of reagent or solvent /test	Instrument cost	Simplicity of procedure	Suitable for on-site	
1	Ultrasonic extraction/filtration/ pre-concentration (using a rotary evaporator)	10 g	250 mL ethanol	50 °C	30 min	UV-spectrophotometry (with internal standard calibration)	No need	Spectrometer	10–100 µg/mL	N/A	N/A	N/A	N/A	N/A	No need	+	+	+	[35]
2	Ultrasonic/decolorization (using charcoal)/filtration	80 mg	50 mL 95% ethanol	N/A	> 30 min	VIS-spectrophotometry	Kedde's reagent	Spectrometer	12–72 µg/mL	1.2 µg	4.23 µg	3–5 min	12	200 µL	100 µl	++	++	++	[36]
3	Ultrasonic/filtration/decolorization (using charcoal)	1 g	20 ml 95% ethanol	N/A	>30 min	FIA	Kedde's reagent	Spectrometer	5.0–150.0 µg/mL	1.5 µg/mL	N/A	1.2 min	50	200 µl	1 mL/min	++	++	+++	[37]
4	Simple extraction/centrifuge	20 g	500 mL ethanol	Room temp.	N/A	VIS-spectrophotometry	Kedde's reagent	Spectrometer	N/A	N/A	N/A	10 min	6	2 mL	0.5 mL	+++	++	++	[38]
5	Ultrasonic/filtration/decolorization (refluxed with charcoal)	10 g	250 ml 95% ethanol	40° C	> 60 min	VIS-spectrophotometry	Kedde's reagent	Spectrometer	10–50 µg/mL	2.14 µg/mL	6.49 µg/mL	N/A	N/A	1 mL	2 mL	++	+	++	[39]
6	Simple extraction/pre-concentration	1 g	200 mL hot methanol, 75 mL toluene, 200 mL ethyl acetate	N/A	N/A	VIS-spectrophotometry	Baljet's reagent	Spectrometer	5–50 µg/mL	N/A	N/A	20 min	3	200 uL	10 mL	+++	++	+	[40]
7	Reflux extraction/filtration/ precipitation(gelatin)charcoal powder	250 g	3 L 85% ethanol	High temp. for reflux	> 180 min	HPLC	No need	UV detector	0.01–0.5 mg/mL	7.2 µg/mL	21.9 µg/mL	30 min	2	10 µL (injection volume)	1.5 mL/min	+	++	+	[30]
8	Ultrasonic extraction/filtration	250 mg	2.5 mL methanol	N/A	>30 min	HPLC	No need	UV detector	5–200 µg/mL	N/A	N/A	20 min	3	1 uL(injection volume)	0.23 mL/min	++	+++	++	[31]
9	Ultrasonic extraction/filtration, pre-concentration (by evaporation), purification	10 g	methanol	Room temp	>1 h	HPLC	No need	UV detector	50–200 µg/mL	N/A	10 µg/mL	9 min	6	N/A	3 mL/min	+	+	+	[32]
10	Simple extraction (by using Rugged Rotator)/centrifugation/ filtration	0.1 g	4 mL methanol, re-extraction with methanol	N/A	> 30 min	HPLC	No need	UV detector	0.5–1000 µg/mL	0.1 µg/mL	0.25 µg/mL	<18 min	6	5 µL (injection volume)	1.2 mL/min	+	++	+	[33]
11	Ultrasonic extraction/filtration/pre-concentration (by evaporation)	10 g	100 mL ethanol	N/A	>1 h	FT-IR	KBr	FT-IR spectrometer	140–825 µg (in KBr disc)	1.5 µg (in KBr disc)	15 µg (in KBr disc)	20 min	3	N/A	N/A	+	++	+	[41]
12	Simple extraction/filtration	1 g	10 mL methanol	Room temp.	>15 min	colorimetry	Kedde's reagent	Smartphone	15–100 ug/mL	5 ug/mL	15 ug/mL	3 min	120 (in duplicate)	150 µL	150 µL	+++	+++	+++	This work

pharmaceuticals

Table S2. Accuracy and precision (repeatability and intermediate precision) for total lactone.

Blind samples (µg/mL)	Repeatability (n=3)						Intermediate precision (n=9)	
	Day 1		Day 2		Day 3			
	%Recovery	%RSD	%Recovery	%RSD	%Recovery	%RSD	%Recovery	%RSD
20	96	6	94	2	97	3	96	4
40	100	2	98	5	99	2	99	3
80	101	2	99	4	98	1	99	2
90	101	2	100	1	102	1	101	2

Table S3. Average percentage recoveries obtained from AG standard solutions (25 µg/mL) spiked with interfering compounds.

Concentration of interfering compound (µg/mL)	Recovery (%)							
	Alkaloid		Flavonoid		Phenolic compound			
	Quinine	Caffeine	Rutin	Catechin	Caffeic acid	Gallic acid	Ellagic acid	Tannic acid
5	91 ± 4	90 ± 4	95 ± 5	91 ± 1	92 ± 1	98 ± 3	93 ± 3	105 ± 1
12.5	94 ± 2	95 ± 1	94 ± 3	83 ± 2	93 ± 3	95 ± 3	103 ± 2	105 ± 3
25	98 ± 2	100 ± 2	95 ± 1	70 ± 2	108 ± 2	83 ± 1	103 ± 3	104 ± 5
